# Genome-wide miRNA response to anacardic acid in breast cancer cells

**DOI:** 10.1371/journal.pone.0184471

**Published:** 2017-09-08

**Authors:** David J. Schultz, Penn Muluhngwi, Negin Alizadeh-Rad, Madelyn A. Green, Eric C. Rouchka, Sabine J. Waigel, Carolyn M. Klinge

**Affiliations:** 1 Department of Biology, University of Louisville, Louisville, Kentucky, United States of America; 2 Department of Biochemistry & Molecular Genetics, University of Louisville School of Medicine, Louisville, Kentucky, United States of America; 3 Bioinformatics and Biomedical Computing Laboratory, Department of Computer Engineering and Computer Science, Louisville, Kentucky, United States of America; 4 Department of Medicine, University of Louisville School of Medicine, Louisville, Kentucky, United States of America; Institut de Pharmacologie Moleculaire et Cellulaire, FRANCE

## Abstract

MicroRNAs are biomarkers and potential therapeutic targets for breast cancer. Anacardic acid (AnAc) is a dietary phenolic lipid that inhibits both MCF-7 estrogen receptor α (ERα) positive and MDA-MB-231 triple negative breast cancer (TNBC) cell proliferation with IC_50_s of 13.5 and 35 μM, respectively. To identify potential mediators of AnAc action in breast cancer, we profiled the genome-wide microRNA transcriptome (microRNAome) in these two cell lines altered by the AnAc 24:1n5 congener. Whole genome expression profiling (RNA-seq) and subsequent network analysis in MetaCore Gene Ontology (GO) algorithm was used to characterize the biological pathways altered by AnAc. In MCF-7 cells, 69 AnAc-responsive miRNAs were identified, *e*.*g*., increased let-7a and reduced miR-584. Fewer, *i*.*e*., 37 AnAc-responsive miRNAs were identified in MDA-MB-231 cells, *e*.*g*., decreased miR-23b and increased miR-1257. Only two miRNAs were increased by AnAc in both cell lines: miR-612 and miR-20b; however, opposite miRNA arm preference was noted: miR-20b-3p and miR-20b-5p were upregulated in MCF-7 and MDA-MB-231, respectively. miR-20b-5p target *EFNB2* transcript levels were reduced by AnAc in MDA-MB-231 cells. AnAc reduced miR-378g that targets *VIM* (vimentin) and *VIM* mRNA transcript expression was increased in AnAc-treated MCF-7 cells, suggesting a reciprocal relationship. The top three enriched GO terms for AnAc-treated MCF-7 cells were B cell receptor signaling pathway and ribosomal large subunit biogenesis and S-adenosylmethionine metabolic process for AnAc-treated MDA-MB-231 cells. The pathways modulated by these AnAc-regulated miRNAs suggest that key nodal molecules, *e*.*g*., Cyclin D1, MYC, c-FOS, PPARγ, and SIN3, are targets of AnAc activity.

## Introduction

microRNAs (miRNAs) are ~ 22 nt noncoding RNAs that basepair with complementary sequences in the 3’UTR of their target mRNAs within the RNA-induced silencing complex (RISC) resulting in translational repression and, in many cases, degradation of the target transcript [1]. The selection of the miR-5p or miR-3p arm for inclusion into the RISC complex for 3’-UTR mRNA target selection is determined by the AGO protein [2]. Each miRNA can have hundreds of gene targets resulting in coordinate regulation of cellular pathways [3]. Dysregulated miRNAs in breast cancer contribute to aberrant regulation of cell cycle, differentiation, metabolism, and cancer stem cell (CSC) survival (reviewed in [1, 4–10]).

Anacardic acid (AnAc) is a collective term for the mixture of 6-alkylbenzoic acid congeners that are produced in a number of plants [11]. AnAc has a variety of activities including inhibition of histone acetyltransferase (HAT) activity (reviewed in [12]). Previously, we reported that a specific congener AnAc 24:1n5 acts as a nuclear receptor alternate site modulator (NRAM) to inhibit breast cancer cells in an estrogen receptor (ER)-dependent manner by interfering with ER-DNA binding [13]. In addition, AnAc 24:1n5 also inhibited the growth of MDA-MB-231 triple negative breast cancer (TNBC, *i*.*e*., ERα negative, progesterone receptor negative, and ERBB2 negative) cells, albeit at a higher IC_50_ and through an undefined mechanism [13]. Thus, we hypothesize that additional molecular targets, including miRNAs, are affected by AnAc in breast cancer cells. High Throughput Sequencing (HTS) provides a comprehensive overview of biological processes and pathways affected by AnAc; thus, offering novel insights into potential mechanisms of action and cellular targets.

The goal of this study was to use RNA-Seq to comprehensively identify alterations in miRNAs in ERα-positive, luminal A MCF-7 and MDA-MB-231 TNBC breast cancer cell lines treated with AnAc 24:1n5. Our results identified common and divergent mRNA transcripts down- or up-regulated by AnAc. The pathways modulated by these miRNAs suggest that key nodal molecules, *e*.*g*., Cyclin D1, SMAD, SP1, MYC, c-FOS, PPARγ, BCL2, FOXO3A, MDA2, and SIN3, are targets of AnAc activity.

## Materials and methods

### Materials

AnAc 24:1n5 was purified to greater than 95% as previously reported [13, 14]. For our experiments, AnAc 24:1n5 (AnAc) was dissolved in ethanol (EtOH); thus, EtOH was used as a vehicle control.

### Cell culture and treatments

MCF-7 and MDA-MB-231 cells were purchased from American Type Tissue Collection (ATCC, Manassas, VA). Cells were used at less than 9 passages from ATCC. MCF-7 and MDA-MB-231 cells were maintained in IMEM (Cellgro, Manassas, VA) containing 5% fetal bovine serum (FBS, Atlanta Biologicals, Lawrenceville, GA) and 1% Penicillin/Streptomycin (Cellgro). Cells were grown in phenol red-free IMEM (ThermoFisher) medium containing 5% dextran coated charcoal (DCC)-stripped FBS (hormone-depleted medium) for 48 h prior to treatment with established IC_50_ concentrations of AnAc 24:1n5: 13.5 μM for MCF-7 and 35.0 μM for MDA-MB-231 cells [13] for 6 h and was replicated in three separate experiments.

### RNA isolation and RNA seq

RNA was isolated from MCF-7 and MDA-MB-231 breast cancer cells using the Exiqon miRCURY^™^ RNA Isolation kit (Woburn, MA, USA). RNA concentration was assessed using a NanoDrop spectrophotometer.

### For miRNA RNA-seq

The Truseq Small RNA kit (Illumina) was used to prepare miRNA libraries from 1 μg total RNA. Each Library was individually gel purified on a Novex TBE 6% gel and resuspended in 10uL 10mM Tris-Cl, pH 8.5. Libraries were validated and quantitated by running 1μL on the Agilent Technologies 2100 Bioanalyzer DNA High Sensitivity Chip. 36-cycle single sequencing reads were generated on the Illumina NextSeq500 instrument utilizing the 500 Mid-output v2 (75 cycle) sequencing kit. The resulting samples were divided into 48 FASTQ [15] single-end raw sequencing files representing four conditions: MCF-7 control, MCF-7 treated with AnAc 24:1n5 (MCF-7 AnAc), MDA-MB-231 control, and MDA-MB-231 treated with AnAc 24:1n5 (MDA MB-231 AnAc). These raw data of our RNA-seq are available at Gene Expression Omnibus (GEO) database: accession number GSE78011.

### Differential miRNA expression analysis

A total of three biological replicates for each treatment were analyzed, with four flow cell lanes per replicate. Raw sequence data files were downloaded from Illumina’s BaseSpace (https://basespace.illumina.com/) onto the KBRIN server for analysis the miRDeep2 [16] and edgeR [17]. Each of the four single-end raw. FASTQ files for each replicate (representing the four flow cells) was concatenated into one single-end. FASTQ file using the unix cat command.

Quality control (QC) of the raw sequence data was performed using FastQC (version 0.10.1) [18]. The FastQC results indicated sequence trimming was not necessary since the minimum quality value for all samples was well above Q30 (1 in 1000 error rate) (data not shown).

Given that this is a miR sequencing project, preliminary adapter trimming was performed on each of the samples using a custom file adaptersToTrim.fa which contains a subset of the Illumina TruSeq Small RNA adapter and primer sequences taken from https://support.illumina.com/content/dam/illumina-support/documents/documentation/chemistry_documentation/experiment-design/illumina-adapter-sequences_1000000002694-00.pdf

Sequences were trimmed of the adapters with Trimmomatric v0.33 [19].

The trimmed sequences were directly aligned to the human hg19 reference genome assembly using the mapper.pl wrapper of the miRDeep2 package (v 0.0.7) [16]. This script used bowtie (version 1.1.1) [20], generating alignment files in arf format. The aligned sequences were then used as inputs into the miRDeep2 package and the script quantifier.pl. In addition, this script used the mirBase release 21 [21] mature miRNA and miRNA hairpin sequences downloaded from ftp://mirbase.org/pub/mirbase/CURRENT/. The result was a file containing the number of reads mapping to each of the 2,822 human (hsa) miRs for the specific sample. After quantification, the resulting counts for each miR in each sample were combined into a reads matrix. This was accomplished using a custom perl script, createReadMatrix.pl. Differentially expressed miRs were determined using edgeR [17] and a customized R script, Schultz-Klinge.miRNA.R. Using a p-value cutoff of 0.05, the number of differentially expressed miRs in each comparison is shown in Table 1.

**Table 1:** Differentially expressed miRNAs (DEmiRs). The log2-fold change with zero value in the control conditions was arbitrarily set to one and the maximum log2-fold change value and those with zero value in the treatment conditions were arbitrarily set to the minimum log2-fold change value of minus one. The number of differentially expressed genes in each comparison is shown and the number of upregulated genes indicated with the upward arrow and downregulated genes indicated by downward arrow.

Comparison	Cutoff	Number of DEmiRs
MCF-7 AnAc *vs*. control	P ≤ 0.05	69 (**↑48**, **↓21**)
MDA-MB-231 AnAc *vs*. control	P ≤ 0.05	37 (**↑15**, **↓22**)
All Cells AnAc *vs*. All Cells control^z^	P ≤ 0.05	25 (**↑13**, **↓12**)
All MCF-7 *vs*. All MDA-MB-231 control^y^	P ≤ 0.05	795 (**↑510**, **↓285**)

^Z^ All Cells is the sum of both cell lines

^Y^ Sum of AnAc treatment and control for each cell line

### *In silico* network analysis

We performed pathway and network analysis of differentially expressed genes in MetaCore^™^ version 6.27 (GeneGO, Thomson Reuters, New York, N.Y.). MetaCore^™^ is a web-based software suite for multiple applications in systems biology including RNA-seq analysis as used here. MetaCore^™^ analyses are based on MetaBase (http://metadatabase.org/), a 100% manually-curated integrated database of mammalian biology that contains over 6 million experimental findings on protein-protein, protein-DNA, protein-RNA, and protein-compound interactions; metabolic and signaling pathways; and other information [22].

**Fig 1:**
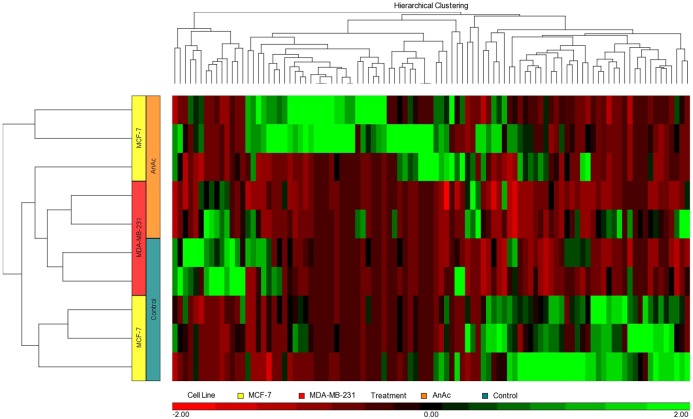
Heat map of miRNAs significantly altered in AnAc-treated MCF-7 and MDA-MB-231 cells. miRNAs significantly affected by AnAc were analyzed using Partek Genomic Suite^™^ to generate the heat map.

Generation of heatmaps: Files of miRNAs significantly altered by AnAc treatment in each cell line were imported into Partek software Version 6.6 (Partek Inc., St Louis, MO.) and Partek Genomic Suite^™^ was used to generate heatmaps (Fig 1, S1 and S2 Figs). Each hierarchical clustering was created using Euclidean distance as similarity measure for genes and samples. We noted that one of the three MCF-7 AnAc samples appeared to behave as a hybrid between the other two AnAc treated and three control (EtOH)-treated samples (S2 Fig).

### RNA isolation, RT-PCR and quantitative real-time PCR (qPCR) of miRNAs and mRNAs

Cell growth, treatment and RNA isolation and quantification/quality assessment were performed as described above. For miRNA, RNA was converted to cDNA using the Taqman^®^ miRNA Reverse Transcription kit (PE Applied Biosystems). For mRNA, RNA was converted to cDNA using the High Capacity cDNA Reverse Transcription kit (PE Applied Biosystems). Primers for hsa-miR-268g, hsa-miR-612, hsa-miR-20b-5p, and hsa-miR-20b-3p were purchased from TaqMan (Advanced miRNA assays) and RNU48 (TaqMan) was used as the reference for normalization [23]. Primers for *VIM* (Vimentin) [24]: Forward 5'-GACAATGCGTCTCTGGCACGTCTT-3'; Reverse 5'- TCCTCCGCCTCCTGCAGGTTCTT-3'; for *ZFP36L1* (ZFP36 Ring Finger Protein Like 1, aka ERF1 and BRF1) [25]: Forward, 5′-AGGATGACCACCACCCTCGTGTCT-3′, Reverse, 5′-CCC CCTGCACTGGGAGCACTA-3′, and for GAPDH [26] were purchased from IDT. qPCR was performed using ABI Viia 7 (Life Technologies) with each reaction run in triplicate. The comparative threshold cycle (Ct) method (2^-ΔΔCT^) was used to determine fold change relative to vehicle treated or control transfected cells [27].

### Transient transfection

MCF-7 and MDA-MB-231 cells were transiently transfected for 24 h with miR-612 mimic, miR-612 inhibitor, Anti-miR ^™^ negative control #1, or mirVANA^™^ miRNA mimic negative control #1 (all from Ambion, Life Technologies, Thermo Fisher Scientific, Carlsbad, CA, USA), using Lipofectamine RNAiMAX transfection reagent (Invitrogen, Thermo Fisher Scientific) and Opti-MEM^®^ Reduced Serum Medium (Invitrogen, Thermo Fisher Scientific). After 24 h of transfection, cells were treated with ethanol (EtOH, vehicle control) or 13.5 or 35 μM AnAc, for MCF-7 and MDA-MB-231 respectively, in phenol red-free IMEM medium containing 5% DCC-stripped FBS for 48 h prior to MTT assay (CellTiter 96, Promega, Madison, WI, USA). Two separate experiments were performed with quadruplicate wells within each experiment. For analysis of miR-612 expression in transfected cells, the medium was changed 24 h after transfection as above, without any treatment and RNA was harvested (see above) a total of 72 h post transfection, *i*.*e*., at the same time the MTT assay was performed for qPCR of miR-612 using RNU48 as a control (see above).

## Results and discussion

### RNA-seq analysis of AnAc-regulated miRNAs

MCF-7 luminal A (ERα+) and MDA-MB-231 TNBC (triple negative breast cancer) cells were incubated in hormone-depleted medium for 48 h prior to a 6 h treatment with the previously established IC_50_ concentrations of AnAc 24:1n5 for MCF-7 (13.5 μM) and MDA-MB-231 (35.0 μM) cells [13]. The 6 h time point was selected based on transcriptome studies in MCF-7 cells to identify primary gene targets [28] and because AnAc 24:1n5 has no overt effect on the viability of either MCF-7 or MDA-MB-231 at that time [13, 29]. The goal was to identify early miRNA changes in response to AnAc 24:1n5 in each cell line. For target analysis, only miRNA transcripts that showed a log2 fold-change greater than 1 (or -1 for repressed miRNAs) were included.

**Fig 2:**
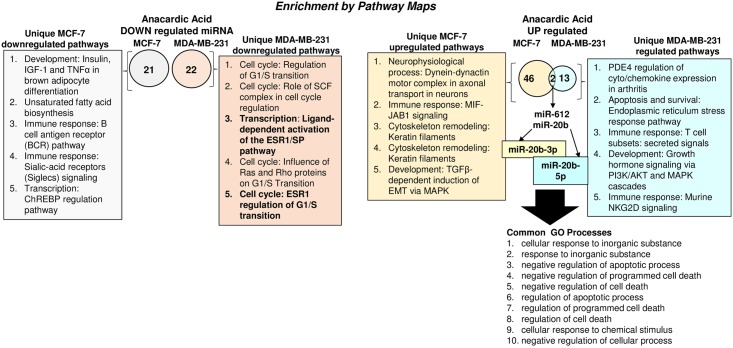
Enrichment analysis of miRNA-seq data. Differentially expressed genes were identified in pairwise comparisons: MCF-7 AnAc vs. MDA-MB-231 AnAc using the tuxedo suite of programs including cufflink-cuffdiff2. The Venn diagrams show the number of common and differentially expressed genes significantly downregulated (A) and upregulated (B). Pathway analysis was performed using GeneGo Pathways Software (MetaCoreTM). The pathways identified for each comparison are listed in the order provided by MetaCoreTM analysis.

Differentially expressed miRNAs (DEmiRs) were identified for four pairwise comparisons (MCF-7 AnAc-treated *vs*. MCF-7 control; MDA-MB-231 AnAc-treated *vs*. MDA-MB-231 control; MCF-7 and MDA-MB-231 AnAc treated *vs*. MCF-7 and MDA-MB-231 control; MDA-MB-231 AnAc treated and control *vs*. MCF-7 AnAc treated and control) using the tuxedo suite of programs including cufflinks and cuffdiff (version 2.2.1) [30, 31]. Significant DEmiRs with fold-change and p values are listed in S1 and S2 Tables. The number of DEmiRs in each comparison is shown in Table 1. Tables 2–5 list the AnAc-regulated miRNAs in MCF-7 and MDA-MB-231 cells, their genomic location and host gene (if applicable), information about their relevance in breast or other cancers and their experimentally verified, *i*.*e*., *bona fide*, targets. The expression of more miRNAs was significantly changed in response to AnAc in MCF-7 cells *vs* MDA-MB-231 cells (Figs 1 and 2). The heatmap shows that MCF-7 and MDA-MB-231 cells have different responses to AnAc with MDA-MB-231 cells showing less change in response to AnAc compared with MCF-7 cells (Fig 1). These data suggest that AnAc selectivity alters miRNA transcript expression in these two cell lines through mostly non-overlapping mechanisms.

**Table 2:** miRNAs upregulated by AnAc in both MCF-7 and MDA-MB-231 cells. The genomic location of each miRNA was identified in miRAD http://bmi.ana.med.uni-muenchen.de/miriad/ [34]. Verified targets are those experimentally validated targets of the indicated miRNA as demonstrated by 3’-UTR luciferase reporter assay. Since many publications do not include whether the 5p or 3p arm of the miRNA was studied, if the sequence of the miRNA was provided, it was searched in miRBase.org to identify which arm was used in the target gene 3’-UTR luciferase reporter assay.

miRNA	Role in breast or other cancers	Verified targets
miR-612	Chr11, intergenic. Tumor suppressor miRNA in HCC tumors, cells and xenograft tumors [35, 36]. Downregulated in colorectal cancer tumors and cells and acts as a tumor suppressor [37].	For 5p: AKT2 [37] SP1 [38]
miR-20b-3p MCF-7	ChrX, encoded by the miR-106a-363 cluster is intergenic [39]. Oncogenic activity, *i*.*e*., stimulates soft agar colony formation in NIH-3T3 cells [39]. Lower expression in taxol-resistant breast tumors and cells [40]. Expression is stimulated by EGR1 [41].	For 3p: ESR1 [42]; EPAS1 [43]; NCOA3 [40]; BRCA1, PTEN [41]
miR-20b-5p MDA-MB-231		For 5p: ARID4A and MYLIP [39]. HIF1A and VEGF [44]; PPARG, BAMBI, CRIM1 [45] EPHB4 and EFNB2 [46]; PTEN [47]; SOS1 and ERK2 [48].

**Table 3:** miRNAs downregulated by AnAc in MCF-7 cells. The genomic location of each miRNA was identified in miRAD http://bmi.ana.med.uni-muenchen.de/miriad/ [34]. Verified targets are those experimentally validated targets of the indicated miRNA as demonstrated by 3’-UTR luciferase reporter assay. Since many publications do not include whether the 5p or 3p arm of the miRNA was studied, if the sequence of the miRNA was provided, it was searched in miRBase.org to identify which arm was used in the target gene 3’-UTR luciferase reporter assay.

miRNA	Role in breast or other cancers	Verified targets
miR-378g	Chr1, host gene LINC01057 [49]. Target of c-Myc [50]. High miR-378 promotes cancer stem cell (CSC) properties, increased cell survival and colony formation; acts as on oncomiR; correlates with increased SOX2 [51]. Induced during adipogenesis by increasing transactivation by C/EBPα and C/EBPβ [52].	VIM [51] TOB2 [50] SUFU and TUSC2 [53] HDAC4 [54]
miR-509-1-3p, -2-3p, -3-3p	miR-509-1, -2, and -3 are in ChrX, host gene LOC107984060. Tumor suppressor miRNA [55, 56]. Inhibited by E_2_ in MCF-7 cells [57]. Anti-metastatic mRNA: The expression of miR-509 was reported to be attenuated in brain metastatic lesions compared to their enrichment in primary breast tumors [58].	For 3p: RHOC [58]; For 5p: YWHAG [59]
miR-513b-5p	ChrX, host gene LOC107984060. Cluster with miR- 506, 507, 208, 509–1,-2,-3, 514b; Acts as a tumor suppressor in gastric cancer cells [60]	For 5p: HMGB3 [60]
miR-548, 548j-5p, 548l	MIR548J: Chr22: host gene HMGB1P10; MIR548L: Chr 11 host gene ANKRD48. miR-548J functions as a metastasis promoter in breast cancer cells [61].	miR-548L: AKT [62]; miR-548j-5p: TNS1 [61]
miR-597-3p	Chr8, host gene TNKS downregulated in colorectal cancer [63]	
miR-1238-3p	Chr19, host gene ARG4D. *no publications in PubMed*	For 3p: LHX2 [64]
miR-1915-3p	Chr10, host gene CASC10. Processing of pri-miR-1915 to pre-miR-1915 is increased by p53 [65].	For 3p: BCL2 [66]
miR-3146	Chr7, host gene TWISTNB. *no publications in PubMed*	
miR-4430	Chr2 intergenic. *no publications in PubMed*	
miR-5002-5p	Chr3, host gene KALRN. *no publications in PubMed*	
miR-5187-5p	Chr2, host gene TOMM40L. *no publications in PubMed*	
miR-6717-5p	Chr14, host gene NDRG2. *no publications in PubMed*	
miR-6773-3p	Chr16, host gene ESRP2. *no publications in PubMed*	
miR-6804-5p	Chr19, host gene PPP6R1. *no publications in PubMed*	
miR-6814-5p	Chr21, host gene RIPK4. *no publications in PubMed*	
miR-6838-5p	Chr7, host gene PLOM. *no publications in PubMed*	
miR-6873-3p	Chr6, host gene WDR46. *no publications in PubMed*	

**Table 4:** miRNAs upregulated by AnAc MCF-7 cells. The genomic location of each miRNA was identified in miRAD http://bmi.ana.med.uni-muenchen.de/miriad/ [34]. Verified targets are those experimentally validated targets of the indicated miRNA as demonstrated by 3’-UTR luciferase reporter assay in the cited reference. Since many publications do not include whether the 5p or 3p arm of the miRNA was studied, if the sequence of the miRNA was provided, it was searched in miRBase.org to identify which arm was used in the target gene 3’-UTR luciferase reporter assay.

miRNA	Role in breast or other cancers	Verified targets
Let-7a-2-3p	Chr11; intergenic. Lower expression metastatic breast tumors [67]. Downregulated by E_2_ treatment in MCF-7 cells [68]. Decreased expression with breast tumor grade and upregulated KEGG pathway targets have roles in cancer-related pathways, including cycle (MCM2), Jak-STAT (SOCS1), MAPK (STMN1), PPAR signaling (ME1) [69]. Transfection of MCF-7 and MDA-MB-231 cells with let-7a mimics inhibits cell proliferation, colony formation, cell migration and invasion and HMGA1 protein [70].	None experimentally validated for 3p.
miR-378j	Chr17, host gene DDX52. *no publications in PubMed*	
miR-450a-1-3p	ChrX, intergenic, clustered with miR-424, 503, 542, 450a-2, and 450b. No publications relating to miR-450a-1 in PubMed, but miR-450a expression was higher in lymph node metastasis in breast cancer [71] and in endometrial carcinosarcomas [72].	None validated for 3p. For 5p: DNMT3a [73]
miR-520a-5p	Chr19, intergenic. miR-520a-3p inhibits proliferation by targeting HOXD8 in non-small cell lung cancer	None experimentally validated for 5p. For 3p: CCND1 and CD44 [74]
miR-520d-5p	Chr19, intergenic. involved in HER2-receptor-related differentiation through undefined mechanisms [75]. Overexpression by lentiviral-miR-520d infection of human HLF and Huh7 hepatoma cells converted the cells to non-tumorigenic and less differentiated normal stem cells, but no miRNA target genes were validated [76]. Acts as a tumor suppressor in colorectal cancer [77].	For 5p: CTHRC1 [77]
miR-548ag-1	Chr4, intergenic. *no publications in PubMed*	
miR-551b-5p	Chr3, intergenic. Downregulated by E_2_ in MCF-7 cells [57]. Down-regulated in aggressive breast tumors [78]. Upregulated in TAM-resistant MCF-7 cells [79]. Upregulated in serum samples from prostate cancer patients compared with benign prostatic hyperplasia patients [80]. Upregulated in recurrent epithelial ovarian cancer (OVCa) [81]. Upregulated in OVCa stem cells, promotes proliferation, invasion, and chemoresistance [82].	None experimentally validated for 5p. For 3p: FOXO3 and TRIM31 [82]
miR-562	Chr2, host gene DIS3L2. Upregulated in serum samples from prostate cancer patients with disseminated disease compared with benign prostatic hyperplasia patients [80].	EYA1 [83]; IL22 [84]
miR-663a	Chr20, intergenic. Upregulated by E_2_ in ECC-1 cells [85]. Transcription increased by ZNF224 [86]. Acts as a tumor suppressor and is downregulated in in gastric [87], colorectal [88], prostate [89], breast [86], hepatocellular [90], pancreatic [91], non-small cell lung cancer [92]. Transcription factor Ets-2 binds the miR-663 promoter and stimulates transcription in prostate cancer cells [89].	TP53 (P53) and CDKN1A (p21) [86] JUND [92] TGFB1 [91] HMGA2 [90]
miR-664b-5p	ChrX, host gene DKC1. Acts as a tumor suppressor in osteosarcoma [93] and as an oncomiR- in T-cell acute lymphoblastic leukemia [94] and cervical cancer [95].	None experimentally validated for 5p. For 3p: FOXO4 [96]; MAT1A [97]; PLP2 [98]; SOX7 [93]
miR-921	Chr1, host gene FAM78B. Downregulated in bladder cancer [99].	CBR1 [100]
miR-1229-5p	Chr 5, host gene MGAT4B. Upregulated in serum of colorectal cancer patients [101]. Overexpressed in breast cancer and correlated with poor prognosis for patients [102].	None experimentally validated for 5p. For 3p; GSK3B, APC and ICAT [102].
miR-1287-3p	Chr10, host gene PYROXD2. Downregulated in MCF-7 cells that are aromatase inhibitor resistant [103]. Hypermethylated in cervical cancer [104], downregulated in larynx carcinoma [105], anaplastic astrocytomas and/or glioblastomas [106].	None experimentally validated for 3p. For 5p: ATF6B [107]
miR-1976	Chr1, host gene RPS6KA1; Acts as a tumor suppressor in NSCLC [108].	PLCE1 [108]
miR-3132	Chr2, host gene TMEM198; *no publications in PubMed*	
miR-3195	Chr20, intergenic; *no publications in PubMed*	
miR-3960	Chr9, intergenic. the lncRNA HOTAIR1 competitively binds to miR-3960 and regulates hematopoiesis [109].	HOXA2 [110]
miR-4436b-1-3p	Chr2, host gene MALL. Appears to be a strong pathogenic candidate in Autism Spectrum Disorders (ASDs) [111].	
miR-4436b-2-3p	Chr2, intergenic. Appears to be a strong pathogenic candidate in ASDs [111].	
miR-4485-5p	Chr11, host gene MTRNR2L8. Is transported into mitochondria and inhibits 16S rRNA processing and mitochondrial protein synthesis [112]. Acts as a tumor suppressor in MCF-7 cells *in vitro* and in MDA-MB-231 cells in xenograft studies in mice [112].	
miR-4516	Chr16, host gene PKD1. Upregulated by fine particulate matter (PM2.5) treatment of A549 NSCLC cells [113]. High expression was associated with infiltrative growth of follicular variant of papillary thyroid carcinomas [114].	STAT3 [115], RPL37 [113]
miR-4634	Chr5, intergenic. One of five miRNAs in serum that detects breast cancer [116]	
miR-4659a-3p	Chr8, host gene AGPAT5. *no publications in PubMed*	
miR-4661-3p	Chr8, host gene LRRC69. miR-466l upregulates both mRNA and protein expression of IL-10 in macrophages by binding to the 3’UTR of IL10 and inhibiting RNA binding protein-induced transcript degradation [117].	
miR-4675	Chr10, intergenic. *no publications in PubMed*	
miR-4687-3p	Chr11, host gene STIM1. *no publications in PubMed*	
miR-4692	Chr11, *no publications in PubMed*	
miR-4695-3p	Chr1, host gene ALDH4A1. *no publications in PubMed*	
miR-4701-3p	Chr12, host gene ADCY6. Downregulated in papillary thyroid carcinoma (PTC) [118].	
miR-4741	Chr18, host gene RBBP8. Downregulated in serum of HCC patients treated with transarterial chemoembolisation (TACE) with bad response to TACE [119].	
miR-4756-5p	Chr20, host gene BCAS1. *no publications in PubMed*	
miR-5008-3p	Chr1, host gene WNT9A. *no publications in PubMed*	
miR-5585-5p	Chr1, host gene TMEM39B. *no publications in PubMed*	
miR-6087	ChrX, intergenic. Identified in human mesenchymal stem cells and downregulated during endothelial differentiation [120]. Upregulated in intermediate monocytes [121].	ENG [120]
miR-6126	Chr16, host gene NAA60. Exosomal tumor suppressor is downregulated in ovarian cancer tumors and is released from ovarian cancer cells [122].	ITGB1 [122]
miR-6131	Chr5, host gene ROPN1L. *no publications in PubMed*	
miR-6515-5p	Chr19, host gene CALR. *no publications in PubMed*	
miR-6726-5p	Chr1, host gene ACAP3. *no publications in PubMed*	
miR-6757-5p	Chr12, host gene TNS2. *no publications in PubMed*	
miR-6813-3p	Chr20, host gene RGS19. *no publications in PubMed*	
miR-6857-5p	ChrX, host gene SMC1A *no publications in PubMed*	
miR-6868-5p	Chr17, host gene EXOC7. *no publications in PubMed*	
miR-6874-5p	Chr7, host gene RNF216. *no publications in PubMed*	
miR-7151-5p	Chr10, host gene CTNNA3. *no publications in PubMed*	
miR-8079	Chr13, intergenic. *no publications in PubMed*	
miR-8089	Chr5, host gene BTNL9. *no publications in PubMed*	

**Table 5:** miRNAs downregulated by AnAc in MDA-MB-231 cells. The genomic location of each miRNA was identified in miRAD http://bmi.ana.med.uni-muenchen.de/miriad/ [34]. Verified targets are those experimentally validated targets of the indicated miRNA as demonstrated by 3’-UTR luciferase reporter assay. Since many publications do not include whether the 5p or 3p arm of the miRNA was studied, if the sequence of the miRNA was provided, it was searched in miRBase.org to identify which arm was used in the target gene 3’-UTR luciferase reporter assay.

miRNA	Role in breast or other cancers	Verified targets
miR-23b-5p	Chr9, host gene C9orf3. OncomiR, induced by c-Myc [123]. Lower expression in MDA-MB-231 than MCF-7 cells [124]. Stimulated by E_2_ in ERβ-transfected MCF-7 cells [125]. Involved in regulation of cytoskeletal remodeling and motility [126, 127]. Primary breast tumor expression of mIR-23b correlates with lung metastasis [128]. Metastatic breast cancer cells in patient bone marrow had increased miR-23b [129]. Increased in MCF-7 cell derived exosomes after docosahexaenoic acid (DHA) treatment [130]. miR-23a is 2.5-fold higher in MDA-MB-231 than MCF-7 cells and downregulates CDH1 resulting in hyperactivation of Wnt/-catenin signaling, EMT, and metastasis [131].	For 5p: PRODH [132]
miR-141-3p	Chr12, intergenic and clustered with miR-200c [133]. Both OncomiR and tumor suppressor miRNA, depending on tissue-type. Expression is repressed by ZEB1 [134], PELP1 [135], PLK1, KLF8 [136], and progesterone [137, 138] and upregulated by p53 [139]. Downregulated in metastatic breast cancer [71] and in basal-like primary tumors [140]. Expression stimulated by treatment of MDA-MB-231 cells with DNA demethylating agent 5-AZA-CdR [141]. Low circulating miR-141 was associated with lower overall survival of breast cancer patients [142, 143]. Overexpression of mIR-141 stimulates brain metastasis in mouse models and high serum miR-141 levels were associated with shorter brain metastasis–free survival in human breast cancer patients [144]. miR-141 expression is higher in docetaxel-resistant breast cancer cell lines [145].	For 3p: PGR [137]; CTNNB1 [146]; EIF4E [145]; ANP32E [140]
miR-499a-5p	Chr12, host gene MYH7B. SNP rs3746444 G miR-499A>G was associated with increased breast cancer risk in Chinese population [147].	For 5p: IFNAR1 [148]
miR-664b-5p	ChrX, host gene DKC1. No references were found in PubMed.	
miR-1247-5p	Chr14, in the DLK1-DIO3 genomic imprinted microRNA cluster [149]. Downregulated in aromatase-resistant MCF-7 breast cancer cells [103] and lung adenocarcinomas [150]. Acts as a tumor suppressor in pancreatic cancer [151]. Silenced by DNA methylation in lung adenocarcinomas and cell lines and overexpression promotes apoptosis and inhibits cell invasion and migration [152]. Overexpressed in castration-resistant prostate cancer [153].	For 5p: NRP1 and NRP2 [151]; SOX9 [154]; MYCBP2 [153]; MAP3K9 [155]; STMN1 [152]
miR-1273g-3p	Chr1, host gene SCP2. *no publications in PubMed*	
miR-1277-3p	ChrX, host gene WDR44. *no publications in PubMed*	For 3p: LPL [156]
miR-3611	Chr10, host gene CUL2. *no publications in PubMed*	
miR-3614-3p	Chr17, host gene TRIM25. *no publications in PubMed*	
miR-4284	Chr7, host gene STX1A. Stimulated by treatment of primary human glioblastoma cells with a synthetic berbamine derivative [157]. Downregulated in clear cell papillary renal cell carcinoma [158].	
miR-4451	Chr4, host gene ARHGAP24. *no publications in PubMed*	
miR-4743-5p	Chr18, host gene CTIF. *no publications in PubMed*	
miR-5684	Chr19, intergenic. *no publications in PubMed*	
miR-5696	Chr2, intergenic. *no publications in PubMed*	
miR-6126	Chr16, host gene NAA60. Expression is downregulated in ovarian tumors and miR-6126 acts as a tumor suppressor miRNA in ovarian cancer cells [159].	ITGB1 [159]
miR-6513-3p	Chr2, host gene PNKD. *no publications in PubMed*	
miR-6720-5p	Chr6, host gene FOXF2. Upregulated by *Alternaria* spp mycotoxin alternariol (10 μM) treatment of HepG2 cells [160].	
miR-6765-3p	Chr14, host gene JAG2. *no publications in PubMed*	
miR-6796-3p	Chr19, host gene PLD3. *no publications in PubMed*	
miR-6797-5p	Chr19, host gene RPS19. *no publications in PubMed*	
miR-6850-3p	Chr8, host gene RPL8. *no publications in PubMed*	
miR-7109-5p	Chr22, host gene PISD. *no publications in PubMed*	

As shown in the Venn Diagrams of Fig 2, there were no common downregulated miRNAs in AnAc-treated MCF-7 and MDA-MB-231 cells. Only two miRNA were commonly upregulated by AnAc in both MCF-7 and MDA-MB-231 cells: miR-20b and miR-612 (Fig 2, Table 2). The common GO Processes for upregulated miR-20b and miR-612 were identified by MetaCore^™^ analysis and listed in Fig 2; however no matches between genes/proteins for miR-20b and miR-612 were identified in Pathway Maps by MetaCore analysis. Interestingly, AnAc increased miR-20-3p in MCF-7 and miR-20-5p in MDA-MB-231 cells. This suggests that distinct miR-20b targets would be expected to be regulated in response to AnAc upregulation of miR-20b-3p *versus* miR-20-5p in the two cell lines. The selection of which mature miRNA 5p or 3p arm is dominant is determined by thermodynamic and structural properties of the processed pre-miR-duplex AGO protein (reviewed in [32]). The functional consequences of arm selection are therefore distinct. The exact mechanism of miRNA Induced Silencing Complex (miRISC) assembly remains elusive and includes a human miRNA loading complex containing the ds-pre-miRNA, DICER1, TRBP2 and miRNA-free AGO protein as its components. [33]. Recent studies in Huh7 human hepatoma cells showed that an increase in target genes, *i*.*e*., *SLC7A1* (CAT-1), increased the processing of pre-miR-122 to miR-122, implying that increases in target mRNA levels can promote miRNA biogenesis [33]. Whether this is true for other cells and miRNAs remains to be examined. The MetaCore network enrichment analysis of the miRNAs upregulated in AnAc-treated MCF-7 vs. MDA-MB-231 cells identified “Cellular response to inorganic substance” as the top GO process (S3A Fig). The network analyses for miR-20b and miR-612 are shown in S3A and S3B Fig.

There is only one previous examination of miRNAs, mRNAs, and lncRNAs in MCF‐7 and MDA‐MB‐231 cells, but that study used a microarray expression profiling [167] rather than an unbiased RNA-sequencing approach. None of the AnAc-regulated miRNAs was among the miRNAs more highly expressed in MCF-7 compared with MDA-MB-231 cells [167]. In contrast, miR-4284 was more highly expressed in MDA-MB-231 cells [167] and we observed that AnAc decreased miR-4284 in MDA-MB-231 cells (Table 5). The role of miR-4284 in breast cancer is unknown and there are no validated targets of miR-4284, although microRNA.org lists 7,891 putative targets.

### miRNAs downregulated by AnAc in MCF-7 cells

Twenty-one miRNAs were downregulated by AnAc in MCF-7 cells (Table 3). miRNAs are encoded within a gene (intronic or exonic) or are intergenic (reviewed in [168]). miRNAs can be regulated independently or are cotranscribed with their host gene (reviewed in [8]). To examine if the miRNA host gene was downregulated by AnAc in MCF-7 cells we searched GSE78011. In AnAc-treated MCF-7 cells, six downregulated host genes for downregulated miRNAs were identified: MiR-548j host gene *HMGB1P10*; miR-597 host gene *TNKS*; miR-1915 host gene *CASC10*; miR-3146 host gene *TWISTNB*; miR-5187 host gene *TOMM40L*; and miR-6814 host gene *RIPK4*. Whether AnAc selectively inhibits the transcription of these genes via its p300/PCAF histone acetyltransferase (HAT) inhibitory function [169] remains to be examined. Inhibition of HAT activity would be expected to increase gene expression. Interestingly, AnAc inhibits p300/PCAF histone acetyltransferase (HAT) activity [169] and thus could coordinately downregulate this set of miRNAs and host genes by promoting a more condensed genomic state, but experimentally examining the veracity of the supposition is outside this current study and remains to be examined fully. MetaCore transcription factor (TF) network analysis identified CREB1, FosB, SOX4, TCF7L2 (TCF4), PRDM14, JunD, GATA-3, FRA-1, cFos, JunB, FOXp3, and YY1 as significantly associated with these genes. The ability of AnAc to inhibit the activity of these TFs will also need to be experimentally verified.

A decrease in a miRNA would be expected to result in an increase its target transcript expression. Validated targets of each miRNA were identified in the literature. An important note in searching the literature for miRNA targets is that often, whether the miRNA# is the 3p or 5p arm is not stated. However, if the miRNA sequence is provided in a diagram along with the seed match site in a target mRNA’s 3’-UTR, the miRNA sequence can be identified as either 3p or 5p by entering the miRNA sequence in miRBase.org. Clearly, a miRNA-3p and miRNA-5p will have different targets, and thus potentially different cellular effects. When identified in our RNA seq study, the 3p or 5p arm is indicated.

AnAc reduced miR-378g that targets *VIM* (vimentin) [51] and *VIM* mRNA transcript expression was increased in AnAc-treated MCF-7 cells (GSE78011), suggesting a reciprocal regulation. None of the other validated targets of decreased miRNAs (Table 3) were found among the upregulated mRNA transcripts identified in GSE78011. MetaCore network enrichment analysis did not match any of the downregulated miRNAs and Pathway Maps, GO processes, or Process Networks. Networks identified were 1) miR-509: positive regulation of macromolecule metabolic process; 2) miR-584: regulation of gene expression; 3) miR-509, miR584, MDM2, ERK1/2: positive regulation of gene expression (S4 Fig). Based on their CSC and tumor-promoting activities the AnAc downregulation of miR-378g, miR-548, miR-548j, miR-548l (Table 3) would be expected to contribute to the anti-proliferative activity of AnAc.

### miRNAs upregulated by AnAc in MCF-7

AnAc increased the expression of 48 miRNAs in MCF-7 cells (Table 4). None of the host genes (Table 3) of intronic miRNAs was upregulated by AnAc treatment of MCF-7 cells. None of the validated targets of upregulated miRNAs in AnAc-treated MCF-7 cells (Table 4) were found among the AnAc-regulated mRNA transcripts identified in RNA seq (GSE78011). Given their roles as ‘tumor suppressor’ miRNAs in inhibiting breast and other cancer cell proliferation and activities (see Table 4), the increases in let-7a-2-3p, miR-520a-5p, miR-520d-5p, miR-551b-5p, miR-612, miR-663a, miR-1287-3p, miR-4485-5p, and miR-6126 may play roles in AnAc-mediated inhibition of breast cancer cell proliferation. miR-520a-5p and miR-520d-5p are in a cluster of miR-520 isomers (a-h) on Chr 19 that share the same seed sequence, and thus are predicted to have common targets. miR-520f was recently reported to target *ADAM9*, thus inhibiting internalization of E-cadherin, and *TGFBR2* that inhibits TGFβ signaling–mediated induction of ZEB1/2 and/or SNAI which thus allows CDH1 (E-cadherin) transcription, thus blocking EMT [170].

MetaCore analysis of these miRNAs identified “embryo implantation, cellular response to amino acid stimulus” as the top GO process (S5A Fig). Network analysis identified two top networks: 1) mi-1229-3p, miR-520a-5p, miR-612, miR-4516, miR-562: positive regulation of metabolic process (S5B Fig); and 2) miR 20b-3p, miR 663a, let-7a-5p, miR-1229 -3p, SMAD3: regulation of cell proliferation (S5C Fig). Network analysis of TFs associated with the 48 upregulated miRNAs identified c-Myc, N-Myc, EPAs1, E2F1, SOX2, AML1, RUNX10, NANOG, MITF, EGR1, and ZNF224 in the top ten TFs. Whether AnAc may activate these TFs to increase the transcription of the upregulated miRNAs or selectively increase miRNA stability will require further examination.

### miRNAs oppositely regulated by AnAc in MCF-7 and MDA-MB-231 cells

In contrast, miR-6873 showed opposite AnAc regulation in the two cell lines: it was downregulated in MCF-7 and upregulated in MDA-MB-231 cells (Tables 2 and 5). There are no publications in PubMed on miR-6873 and miR-6873 was not listed in microRNA.org or miRTarBase. Thus, its relevance to AnAc responses in these two cell lines is unknown.

### miRNAs downregulated by AnAc in MDA-MB-231 cells

Twenty-two miRNAs were downregulated by AnAc in MDA-MB-231 cells and none of these overlapped with miRNAs downregulated by AnAc in MCF-7 cells (Table 5). The chromosome location and host gene, if warranted, of each of the AnAc-downregulated miRNAs are identified in Table 5. To examine if the miRNA host gene was downregulated by AnAc in MDA-MB-231 cells, we searched GSE78011. miR-1277 host gene *WDR44* was downregulated by AnAc in MDA-MB-231 cells. WDR44 encodes a protein that interacts with the small GTPase rab11 and is involved in endosome recycling [171]. There are no validated targets for miR-1277 in miRTarBase.

Downregulation of a miRNA would be expected to increase the expression of its targets; hence, we searched our data of mRNAs upregulated by AnAc in MDA-MB-231 cells (550 genes, GSE78011) for the validated targets in Table 5, but none were reciprocally upregulated. This may be because the miRNA and mRNA for RNA seq were extracted at the same time, *i*.*e*., after 6 h of AnAc treatment, or that these mRNAs are not expressed or targeted in MDA-MB-231 cells. Given their roles as putative oncomiRs the downregulation miR-23b and miR-1247 may play a role in the anti-proliferative activity of AnAc in in MDA-MB-231 cells.

Analysis of the data identified *ZFP36L1* as a putative target of miR-3614 in MDA-MB-231 cells. Interestingly, AnAc downregulated miR-3614 and upregulated *ZFP36L1* transcript expression in MDA-MB-231 cells, suggesting an inverse correlation. *ZFP36L1* has been identified as a cancer gene due to mutations in breast cancer and acts in a recessive manner [172]. ZFP36L1 is a member of the TTP family of tandem zinc finger proteins that bind AU-rich elements (AURE) in the 3′-end of target gene transcripts and promote target degradation, *e*.*g*. *STARD1* [173], *VEGFA* [174], *NR4A2* [175], *BCL2* [176], *LDLR* [177], *STAT5B* [178], and *CDK6* [179]. Of these genes, only *VEGFA* and *LDLR* were identified as differentially expressed genes in AnAc-treated cells. *LDLR* was downregulated whereas *VEGFA* was upregulated in AnAc-treated MDA-MB-231 cells. Interestingly, medroxyprogesterone acetate (MPA, a synthetic progestin), but not E_2_, upregulates ZFP36L1 transcription in MCF-7 cells [25].

MetaCore analysis of the AnAc-downregulated miRNAs in MDA-MB-231 cells identified one canonical pathway map: “Development: miRNA-dependent regulation of EMT” and the 10 GO processes in S6A Fig. Network analysis identified two top networks: 1) miR-23b-3p, miR-499, miR-499-3p, miR-499-5p, c-Fos (S6B Fig), and miR-141, miR-141-3p, miR-1247-5p, PPAR-gamma, BMI-1 (S6C Fig).

### miRNAs upregulated by AnAc in MDA-MB-231 cells

Fourteen miRNAs were increased by AnAc-treatment of MDA-MB-231 cells (Table 6). We have described miR-20b-5p and miR-612 upregulation in the context of similar results in AnAc-treated MCF-7 cells (Table 2, Fig 2, S2 Fig). The chromosome location and host gene, if warranted, of each of the AnAc-upregulated miRNAs are identified in Table 6. Interestingly, most of the downregulated miRNAs were intergenic. miR-1298 is in encoded in *HTR2C*, but *HTR2C* was not among the AnAc-regulated genes in MDA-MB-231 cells in GSE78011. An increase in a miRNA would be expected to result in a decrease of its target transcript. miR-20b-5p target EFNB2 (ephrin B2) expression was downregulated in AnAc-treated MDA-MB-231 cells, but none of the validated targets of the upregulated miRNAs (Table 6) were found among the AnAc-downregulated mRNA transcripts identified in RNA seq (GSE78011). Given their roles as ‘tumor suppressor’ miRNAs (see Table 6), the increases in miR-29b, miR-612, and miR-1298 may contribute to the antiproliferative activity of AnAc in MDA-MB-231 cells.

**Table 6:** miRNAs upregulated by AnAc in MDA-MB-231 cells. The genomic location of each miRNA was identified in miRAD http://bmi.ana.med.uni-muenchen.de/miriad/ [34]. Verified targets are those experimentally validated targets of the indicated miRNA as demonstrated by 3’-UTR luciferase reporter assay. Since many publications do not include whether the 5p or 3p arm of the miRNA was studied, if the sequence of the miRNA was provided, it was searched in miRBase.org to identify which arm was used in the target gene 3’-UTR luciferase reporter assay.

miRNA	Role in breast or other cancers	Verified targets
miR-378f	Chr1, intergenic. Downregulated by *E6/E7* silencing in HeLa cells [161].	
miR-1257	Chr20, intergenic. Downregulated in dedifferentiated liposarcoma [162].	
miR-1298-5p	ChrX, host gene HTR2C clustered with miR-764, miR1912, miR1264, miR-1911, and miR-448. Downregulated in neuroglioma [163]. Identified as an inhibitor the growth of KRAS-driven colon cancer cells both *in vitro* and *in vivo* [164].	For 5p: GJA1 [165], PTK2 and LAMB3 [164]
miR-1304-5p	Chr11, intergenic. Downregulated in NSCLC cells [166].	
miR-3116-1	Chr1, host gene PATJ. *no publications in PubMed*	
miR-3139	Chr4, host gene GAB1. *no publications in PubMed*	
miR-3159	Chr11, intergenic. *no publications in PubMed*	
miR-3936	Chr5, intergenic. *no publications in PubMed*	
miR-4473	Chr9, host gene MLLT3. *no publications in PubMed*	
miR-6794-5p	Chr19, host gene MAST1. *no publications in PubMed*	
miR-6873-3p	Chr6, host gene WDR46. *no publications in PubMed*	
miR-7113-5p	Chr11, host gene NDUFS8. *no publications in PubMed*	

MetaCore analysis of these upregulated miRNAs identified “cellular response to inorganic substance” as the top GO process (S7A Fig). MetaCore analysis identified two networks: 1) miR-1257, Bcl-2, PAX6, FOXO3A, and FOXP3; and 2) miR-20b-5p, PPARγ, MDA2, p57, and Sin3A (S7B and S7C Fig).

### qPCR validation of select AnAc-mediated changes in miRNAs

**Fig 3:**
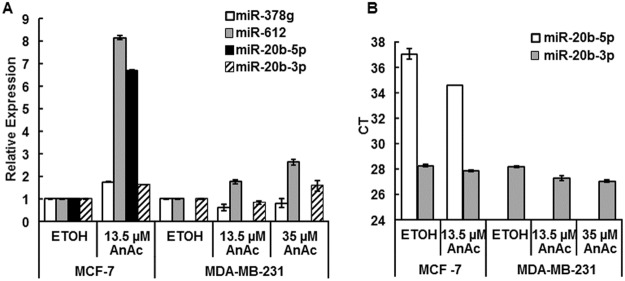
qPCR analysis of select AnAc-regulated miRNA expression. MCF-7 and MDA-MB-231 cells were grown in hormone-depleted medium for 48 h prior to 6 h treatment with 13.5 or 35 μM AnAc. A. qPCR using TaqMan assays for miR-378g, miR-612, miR-20b-5p, and miR-20b-3p was performed using U48 as normalizer. B. CT values for miR-20b-5p and miR-20b-3p expression. miR-20b-5p was not detected in MDA-MB-231 (CT values ‘undetermined). For both A and B: Values are the mean ± SEM of triplicates in one experiment for MCF-7 cells and are the mean ± SEM of two independent experiments for MDA-MB-231 cells.

We selected miR-612, increased by AnAc in both MCF-7 and MDA-MB-231 cells (Table 2); miR-20b-3p and miR-29-5p, upregulated by AnAc in MCF-7 and MDA-MB-231, respectively (Table 2), and miR-378g that was downregulated by AnAc in MCF-7 cells for validation. miR-378g was selected because miR-378g targets *VIM* [51] and *VIM* mRNA transcript expression was increased in AnAc-treated MCF-7 cells (GSE78011), suggesting a reciprocal regulation. Cells were grown in hormone-depleted medium for 48 h prior to 6 h treatment with 13.5 or 35 μM AnAc. As anticipated, AnAc increased miR-612 in both cell lines (Fig 3A). Also as anticipated, AnAc increased miR-20b-3p in MCF-7 cells. We did not detect the anticipated decrease in miR-378g in AnAc-treated MCF-7 cells; however, AnAc reduced miR-378g in MDA-MB-231 cells. We did not detect miR-20b-5p in MDA-MB-231 cells (CT values were undetermined). CT values show that miR-20b-3p is the dominant arm of miR-20b expressed in both cell lines (Fig 3B).

### Effect of altered miR-612 on cell viability

**Fig 4:**
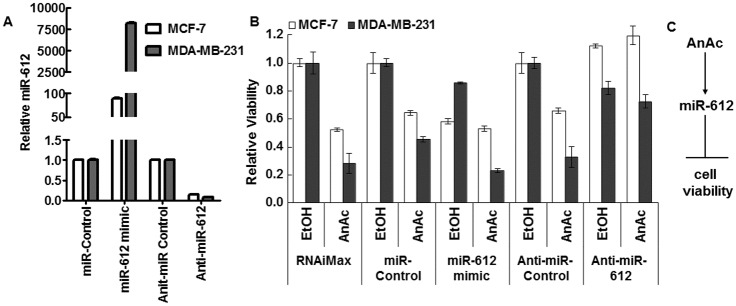
Overexpression of miR-612 inhibits cell viability and inhibition of miR-612 inhibits AnAc’s anti-proliferative activity. MCF-7 and MDA-MB-231 cells were transfected with miR-Control (negative control), miR-612 mimic, anti-miR-Control (negative control), or anti-miR-612 for 24 h prior to 48 h treatment with EtOH (vehicle control) or 13.5 μM (MCF-7) or 35 μM (MDA-MB-231) AnAc. miR-612 expression was measured by qPCR relative to RNU48 in the transfected, untreated cells 72 h after transfection to match the time of the MTT assay (B). Values are the average of triplicate determinations ± SEM in one transfection and are relative to the appropriate transfection control as indicated. Cell viability was evaluated by MTT assay (B). Values for the MTT assay are relative to negative controls and are the avg ± SEM of 2 separate experiments. AnAc is proposed to affect cell viability through miR-612 (C).

Since AnAc increased miR-612 in both MCF-7 and MDA-MB-231 cells (Table 2, Fig 2) and miR-612 has reported tumor suppressor activity in HCC [35, 36] and colorectal cancers [37] (Table 4), we examined how altering miR-612 levels affected cell viability of MCF-7 and MDA-MB-231 cells and their responses to AnAc. Alterations in miR-612 levels in each cell line in response to transfection of miR-612 mimic and anti-miR-612 were demonstrated (Fig 4A). As expected, AnAc inhibited cell viability in both cell lines (Fig 4B). Transfection with miR-612 mimic inhibited cell viability in each cell line with a larger effect in MCF-7 than MDA-MB-231 cells. Transfection with a miR-612 inhibitor had no effect in MCF-7 cells, but inhibited the viability of MDA-MB-231 cells ~ 20%. Notably, the miR-612 inhibitor abrogated the anti-proliferative activity of AnAc in MCF-7 cells and reduced AnAc’s anti-proliferative activity in MDA-MB-231 cells. These results are consistent with a model in which the increase in miR-612 in AnAc-treated MCF-7 and MDA-MB-231 cells plays a role in the anti-proliferative activity of AnAc (Fig 4C).

### qPCR validation of AnAc-mediated changes in mRNAs targeted by miR-378g

**Fig 5:**
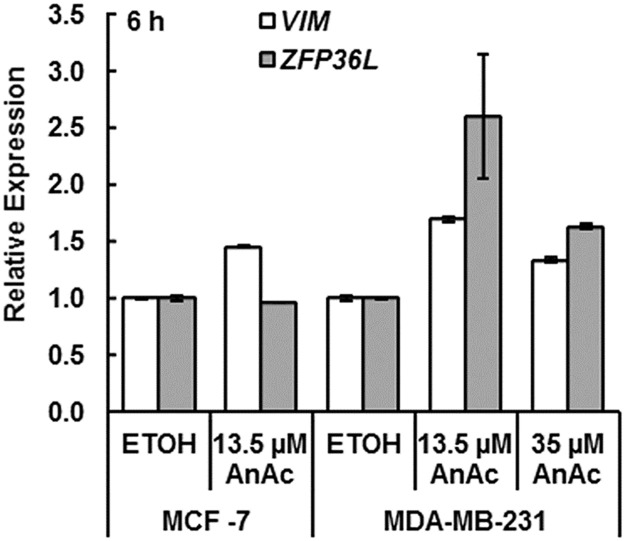
qPCR analysis of mRNA targets of AnAc-downregulated miRNAs. MCF-7 and MDA-MB-231 cells were grown in hormone-depleted medium for 48 h prior to 6 h treatment with 13.5 or 35 μM AnAc. qPCR was performed using GAPDH as normalizer. Values are the mean ± SEM of triplicates in one experiment for MCF-7 cells and are the mean ± SEM of two independent experiments for MDA-MB-231 cells.

We selected *VIM*, a target of miR-378g downregulated by AnAc in MCF-7 cells, and *ZFP36L*, a target of miR-3614 downregulated by AnAc in MDA-MB-231 cells for validation by qPCR. As anticipated from the decrease in miR-378g in RNA seq data (Table 3), we detected a slight increase in *VIM* transcript expression in MCF-7 as well as an increase in *VIM* in MDA-MB-231 cells (Fig 5). However, because qPCR indicated an increase in miR-378g levels in AnAc-treated MCF-7 cells (Fig 5), it is possible that *VIM* is upregulated by AnAc by mechanisms unrelated to miR-378g. In addition, miRNA and mRNA were extracted at the same time, *i*.*e*., after 6 h of AnAc treatment, and it may be that changes in *VIM* mRNA levels require a longer time to be degraded after miR-378g targeting. Transcript levels of *ZFP36L* were increased in AnAc-treated MDA-MB-231 cells (Fig 5), corresponding with the observed downregulation of miR-3614 (Table 5). These data confirm the reciprocal expression of these mRNA transcripts detected in RNA seq and their target miRNAs in the respective AnAc-treated cell line.

### Pathways affected by DEGs and DEmiRs in AnAc-treated MCF-7 cells

MetaCore analysis of DEGs from both mRNA and miRNA data sets of AnAc-treated MCF-7 cells identified NETosis in SLE as the top pathway. The release of neutrophil extracellular traps (NETs) by dying cells (NETosis) was first described as the release of nuclear chromatin, nuclear histones and many granular antimicrobial proteins from neutrophils as one of the first lines of defense against pathogens (reviewed in [180]). The top GO processes were chromatin silencing, negative regulation of gene expression (epigenetic, nucleosome assembly, chromatin assembly, and nucleosome organization. The three gene networks identified were 1): PDEGF PDE6G, APOBEC3H, GGTF II beta, CDIP, p53; 2) miR-499, BMCC1, Histone H1, miR-20b, miR-23b; 3) UCHL1, Protein C, PDK4, EGR1, miR-1298 5p. Network #2 processes include anoikis, negative regulation of fat cell proliferation, regulation of DNA metabolic processes, which reflect the antiproliferative, pro-apoptotic, and NRAM activity of AnAc detected previously in MCF-7 cells [13].

### Pathways affected by DEGs and DEmiRs in AnAc-treated MDA-MB-231 cells

MetaCore analysis of DEGs from both mRNA and miRNA data sets of AnAc-treated MDA-MB-231 cells identified “Immune response, IL-3 signaling via JAK/STAT, p38, JNK, and NFkB” as the top pathway. The top GO processes were “Positive regulation of biological process; cellular response to oxygen-containing compound, positive regulation of cellular process, response to oxygen-containing compound, regulation of developmental process, and response to lipid”. The three gene networks identified were Network #1: Axin, Frizzled, cMyc, WNT, PI3K reg classIA: canonical Wnt signaling pathway, beta-catenin destruction complex disassembly, regulation of cell proliferation, cell surface receptor signaling pathway involved in cell-cell signaling, cell-cell signaling by wnt. Network #2: C/EBPbeta, SOS, NGFR, H-Ras, NGF: positive regulation of cellular metabolic process, positive regulation of MAPK cascade, positive regulation of metabolic process, positive regulation of macromolecule metabolic process, and positive regulation of intracellular signal transduction. Network #3: GALNT4, Keratin80, BCMP101, HEXIM1, PNRC1: translational elongation, translation, amide biosynthetic process, peptide biosynthetic process, peptide metabolic process.

### Conclusions

In summary, we describe the first comprehensive assessment of miRNA expression in response to anacardic acid in ERα+, luminal A MCF-7 and MDA-MB-231 TNBC breast cancer cells. The pathways modulated by these miRNAs suggest that key nodal molecules, *e*.*g*., Cyclin D1, SMAD, SP1, MYC, c-FOS, PPARγ, BCL2, FOXO3A, MDA2, and SIN3, are targets of AnAc activity. In agreement with the pathway analysis, we previously reported that AnAc reduced *CCND1* transcript expression in MCF-7 and MDA-MB-231 cells [13]. The roles of the other proteins and pathways in AnAc responses remains to be investigated.

## Supporting information

S1 Fig Heat map of miRNAs significantly altered in AnAc-treated MCF-7 cells. miRNAs significantly affected by AnAc were analyzed using Partek Genomic Suite^™^ to generate the heat map.
(TIF)

S2 Fig Heat map of miRNAs significantly altered in AnAc-treated MDA-MB-231 cells. miRNAs significantly affected by AnAc were analyzed using Partek Genomic Suite^™^ to generate the heat map.
(TIF)

S3 Fig MetaCore analysis of upregulated miRNAs in AnAc-treated MCF-7 and MDA-MB-231 cells. A) Gene Ontology (GO) processes. The hatched bars are common whereas orange indicates MCF-7 cells. MetaCore Analyze Networks algorithm identified B) miR-20b-5p, Cyclin D1, DEC1 (Stra13), SMAD4 network: circadian regulation of gene expression (41.2%) negative regulation of nucleobase containing compound metabolic process (82.4%), negative regulation of cellular biosynthetic process (82.4%), rhythmic process (58.8%), negative regulation of nitrogen compound metabolic process (82.4%). C) miR-612, SP1, MyCH, gamma-ENaC, DR5 network: muscle filament sliding (36.4%), actin-myosin filament sliding (36.4%), actin filament-based movement (43.2%), muscle contraction (50.0%), actin-mediated cell contraction (36.4%)
(PPTX)

S4 Fig MetaCore analysis of downregulated miRNAs in AnAc-treated cells. MetaCore Analyze Networks algorithm identified A) miR509: B) miR-584, C/EBPbeta, HOX10A; 3) miR-509, miR-584, MDM2, ERK1/2.
(PPTX)

S5 Fig MetaCore analysis of upregulated miRNAs in AnAc-treated MCF-7 cells. A) Gene Ontology (GO) processes. MetaCore Analyze Networks algorithm identified B) miR 1229 3p, miR 520a 5p, miR 612, miR 4516, miR 562: positive regulation of metabolic process (60.5%), negative regulation of apoptotic process (37.2%), negative regulation of programmed cell death (37.2%), negative regulation of cell death (37.2%), viral process (34.9%); C) miR 20b 5p, miR 663a, miR let 7a 5p, miR 1229 3p, SMAD3: regulation of cell proliferation (65.2%), cellular response to growth factor stimulus (43.5%), response to growth factor (43.5%), positive regulation of macromolecule metabolic process (71.7%), response to lipid (52.2%)
(PPTX)

S6 Fig MetaCore analysis of downregulated miRNAs in AnAc-treated MDA-MB-231 cells. A) Gene Ontology (GO) processes. MetaCore Analyze Networks algorithm identified B) miR-23b-3p, miR-499, miR-499-3p, miR-499-5p, c-Fos: response to drug (37.8%), response to abiotic stimulus (48.9%), response to mechanical stimulus (28.9%), cellular response to hormone stimulus (37.8%), response to inorganic substance (37.8%). C) miR-141, miR-141-3p, miR-1247-5p, PPAR-gamma, BMI-1: positive regulation of transcription from RNA polymerase II promoter (76.6%), regulation of transcription from RNA polymerase II promoter (85.1%), positive regulation of nucleic acid-templated transcription (76.6%), positive regulation of transcription, DNA-templated (76.6%), negative regulation of RNA metabolic process (74.5%).
(PPTX)

S7 Fig MetaCore analysis of upregulated miRNAs in AnAc-treated MDA-MB-231 cells. A) Gene Ontology (GO) processes. MetaCore Analyze Networks algorithm identified B) miR-1257, Bcl-2, PAX6, FOXO3A, and FOXP3; and C) miR-20b-5p, PPARγ, MDA2, p57, Sin3.
(PPTX)

S1 Table miRNAs regulated by AnAc in MCF-7 cells. Cells were grown in phenol red-free IMEM (ThermoFisher) medium containing 5% dextran coated charcoal (DCC)-stripped FBS (hormone-depleted medium) for 48 h prior to treatment with established IC_50_ concentrations of AnAc 24:1n5: 13.5 μM for MCF-7 cells [13] for 6 h and was replicated in three separate experiments. Differentially expressed miRNAs (DEmiRs) were identified for pairwise comparisons (MCF-7 AnAc-treated vs. MCF-7 control using the tuxedo suite of programs including cufflinks and cuffdiff (version 2.2.1) Significant DEmiRs with fold-change and p values are listed. These raw data of our RNA-seq are available at Gene Expression Omnibus (GEO) database: accession number GSE78011.
(XLSX)

S2 Table miRNAs regulated by AnAc in MDA-MB-231 cells. Cells were grown in phenol red-free IMEM (ThermoFisher) medium containing 5% dextran coated charcoal (DCC)-stripped FBS (hormone-depleted medium) for 48 h prior to treatment with established IC_50_ concentrations of AnAc 24:1n5: 35.0 μM for MDA-MB-231 cells [13] for 6 h and was replicated in three separate experiments. Differentially expressed miRNAs (DEmiRs) were identified for pairwise comparisons (MDA-MB-231 AnAc-treated vs. MDA-MB-231 control using the tuxedo suite of programs including cufflinks and cuffdiff (version 2.2.1) Significant DEmiRs with fold-change and p values are listed. These raw data of our RNA-seq are available at Gene Expression Omnibus (GEO) database: accession number GSE78011.
(XLSX)
