# Expression of Concern: Genome-wide miRNA response to anacardic acid in breast cancer cells

**DOI:** 10.1371/journal.pone.0356341

**Published:** 2026-08-18

**Authors:** The PLOS One Editors

The *PLOS One* Editors issue this Expression of Concern to inform readers of the following issues:

- References 47, 76, and 94 were retracted after [1] was published.
- Concerns were raised on PubPeer about the following references which the Editors consider may impact the reliability of these articles: 36, 37, 50, 52, 90, 97, 102, 108, 128, 131, 136, 138, and 179. For most of these articles, the concerns were reported after [1] was published.

The corresponding author stated that removal of citations to the retracted articles and articles with concerns raised on PubPeer would have no effect on the data reported in [1] or their interpretation.

Readers are advised to interpret the article [1] with consideration of these issues.
