# Supplementary figures and images for: Genome-wide miRNA response to anacardic acid in breast cancer cells
Source: PLoS One. 2017 Sep 8;12(9):e0184471. doi: 10.1371/journal.pone.0184471 (PMC5590942; doi:10.1371/journal.pone.0184471)

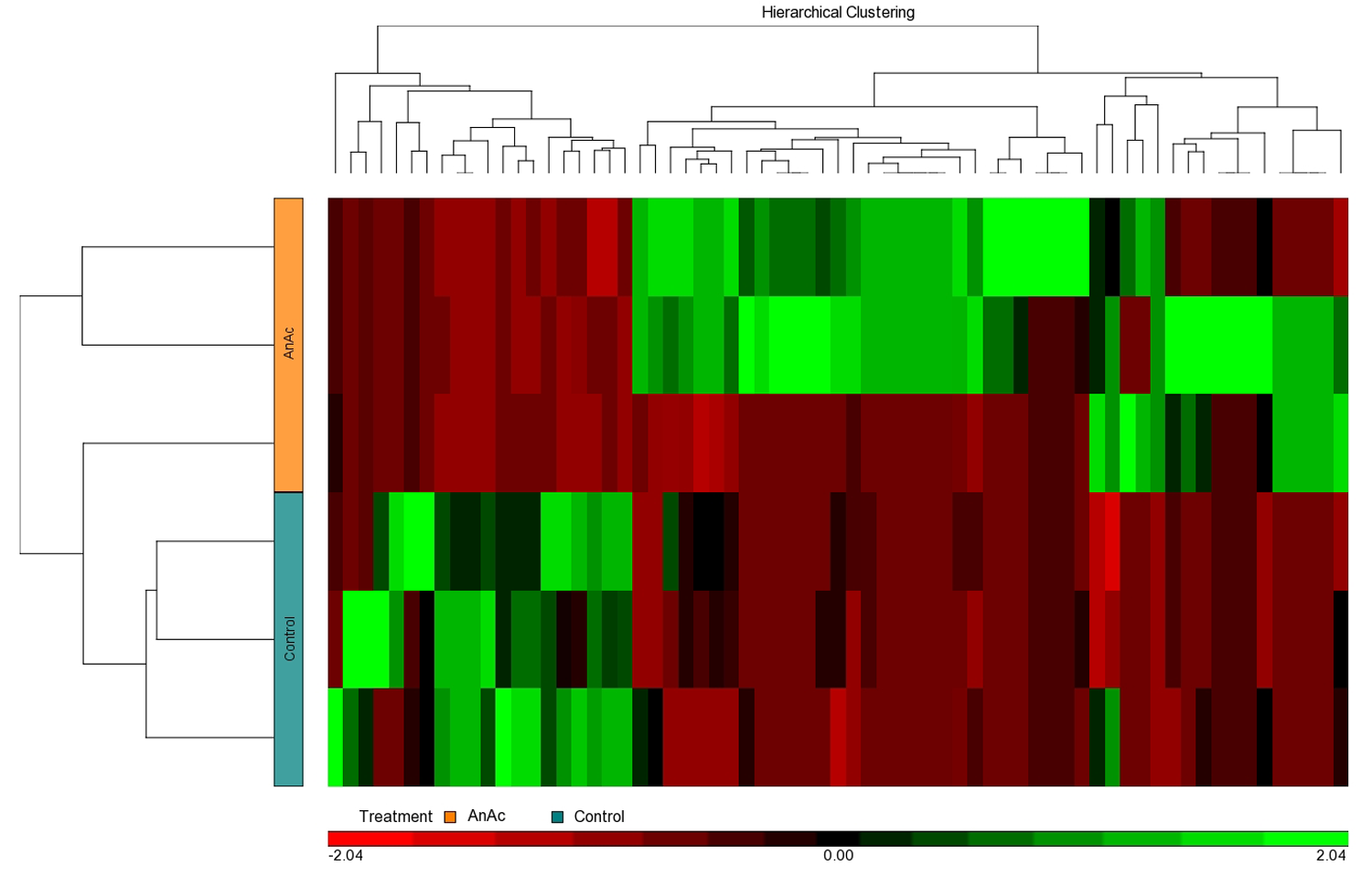

Supplement: S1 Fig — miRNAs significantly affected by AnAc were analyzed using Partek Genomic Suite™ to generate the heat map. (TIF) [file pone.0184471.s001.tif]

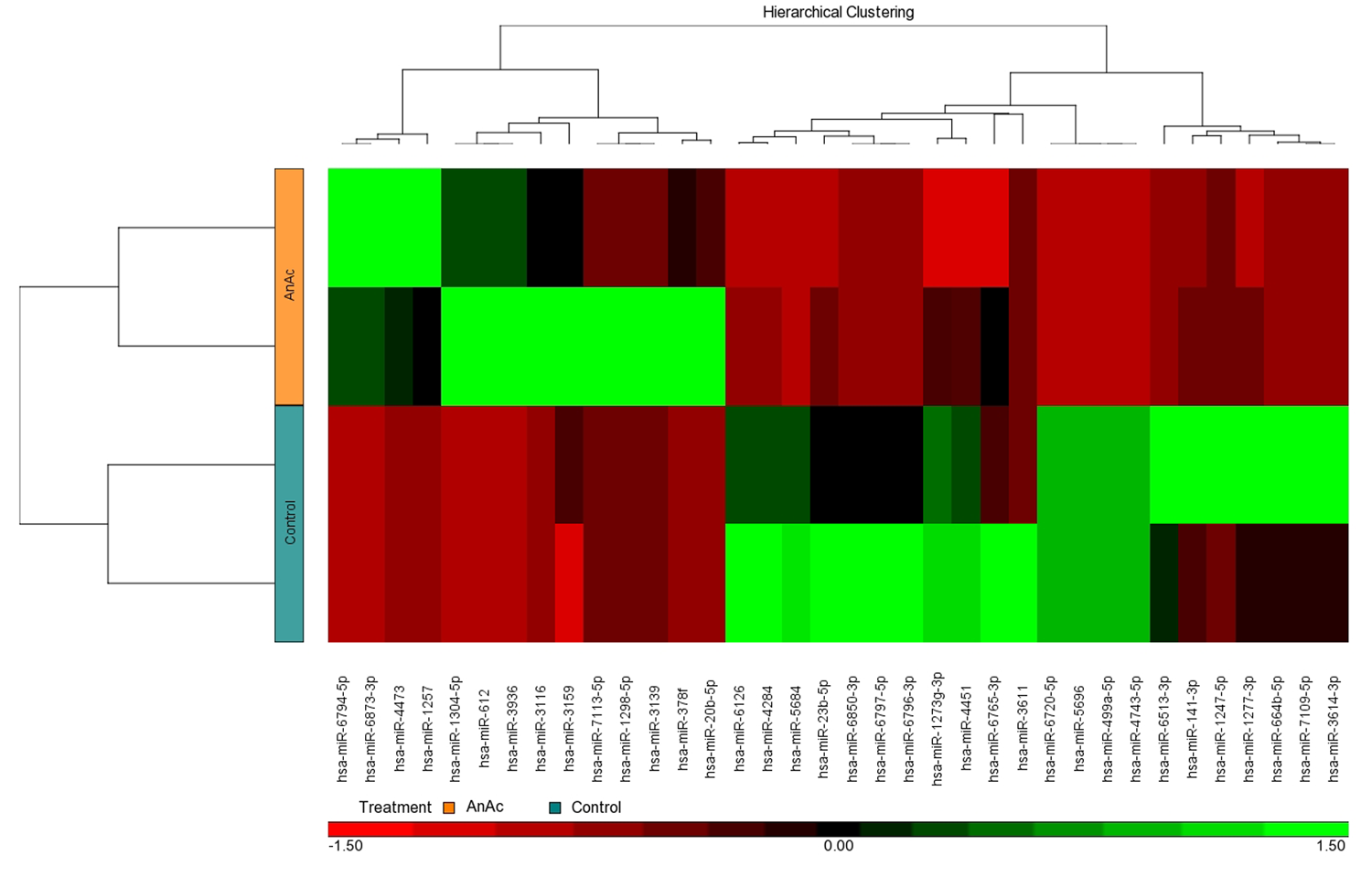

Supplement: S2 Fig — miRNAs significantly affected by AnAc were analyzed using Partek Genomic Suite™ to generate the heat map. (TIF) [file pone.0184471.s002.tif]

## Slide 1
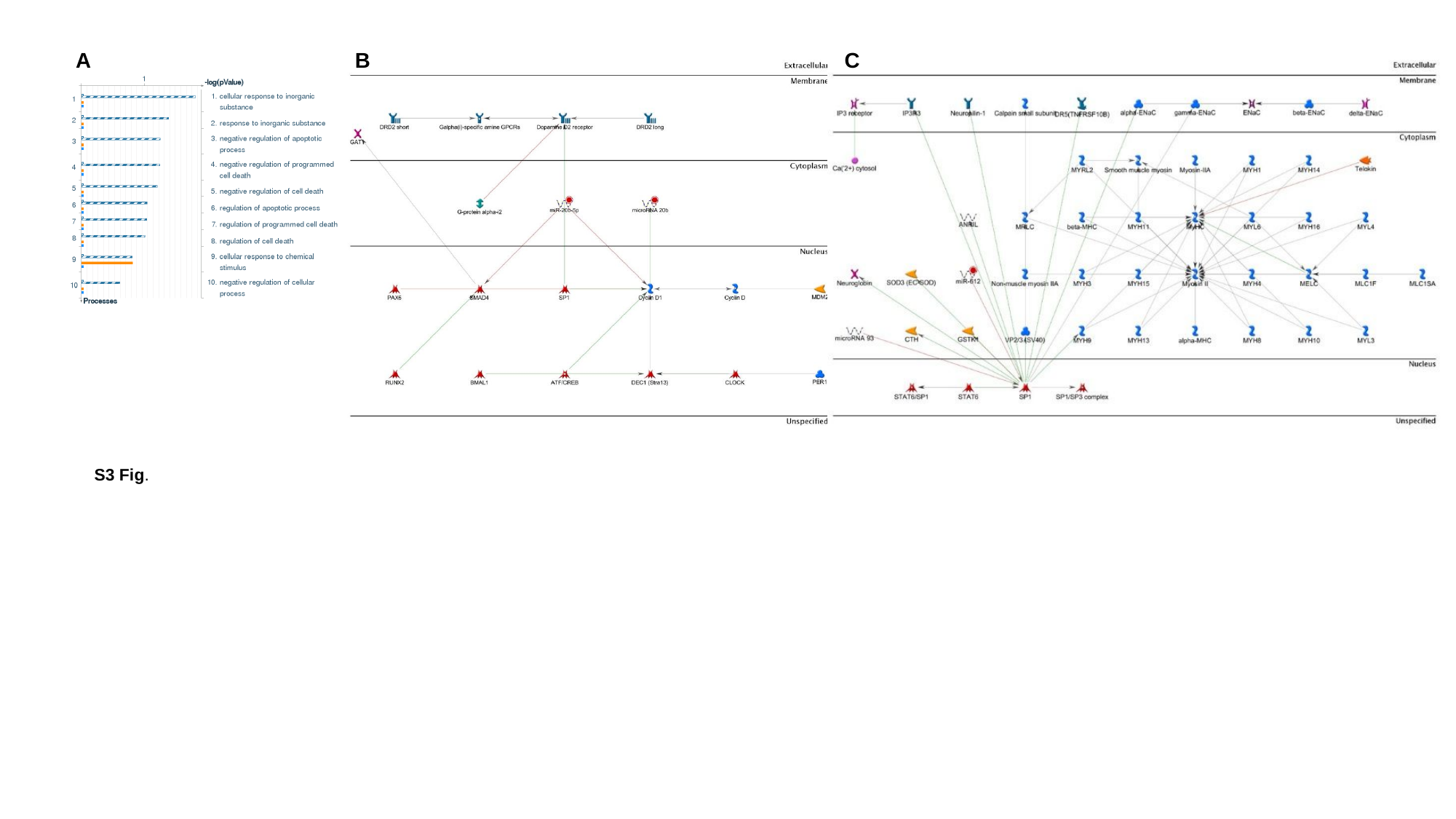

A
B
C
S3 Fig.

Supplement: S3 Fig — A) Gene Ontology (GO) processes. The hatched bars are common whereas orange indicates MCF-7 cells. MetaCore Analyze Networks algorithm identified B) miR-20b-5p, Cyclin D1, DEC1 (Stra13), SMAD4 network: circadian regulation of gene expression (41.2%) negative regulation of nucleobase containing compound metabolic process (82.4%), negative regulation of cellular biosynthetic process (82.4%), rhythmic process (58.8%), negative regulation of nitrogen compound metabolic process (82.4%). C) miR-612, SP1, MyCH, gamma-ENaC, DR5 network: muscle filament sliding (36.4%), actin-myosin filament sliding (36.4%), actin filament-based movement (43.2%), muscle contraction (50.0%), actin-mediated cell contraction (36.4%) (PPTX) [file pone.0184471.s003.pptx]

## Slide 1
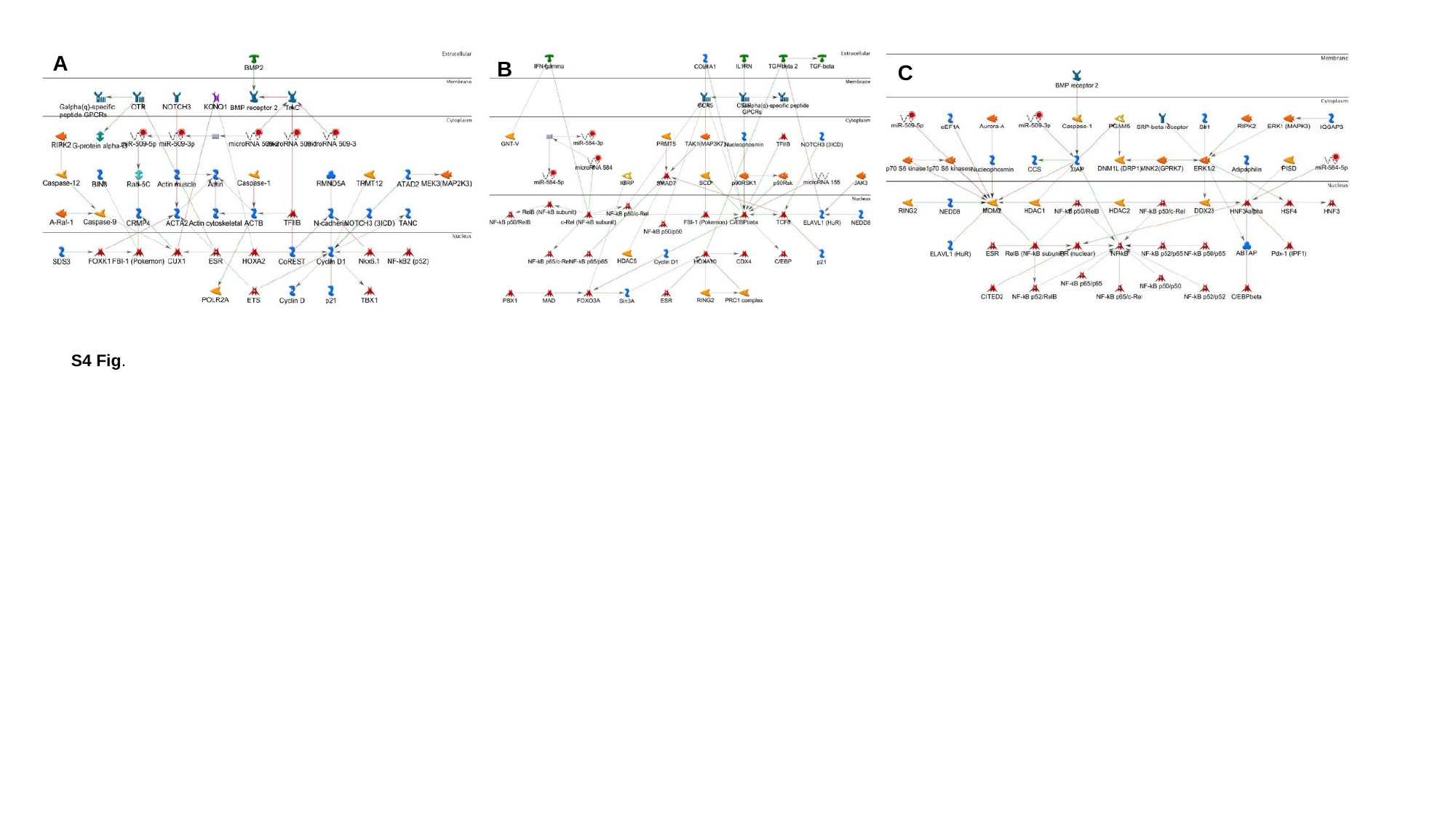

A
B
C
S4 Fig.

Supplement: S4 Fig — MetaCore Analyze Networks algorithm identified A) miR509: B) miR-584, C/EBPbeta, HOX10A; 3) miR-509, miR-584, MDM2, ERK1/2. (PPTX) [file pone.0184471.s004.pptx]

## Slide 1
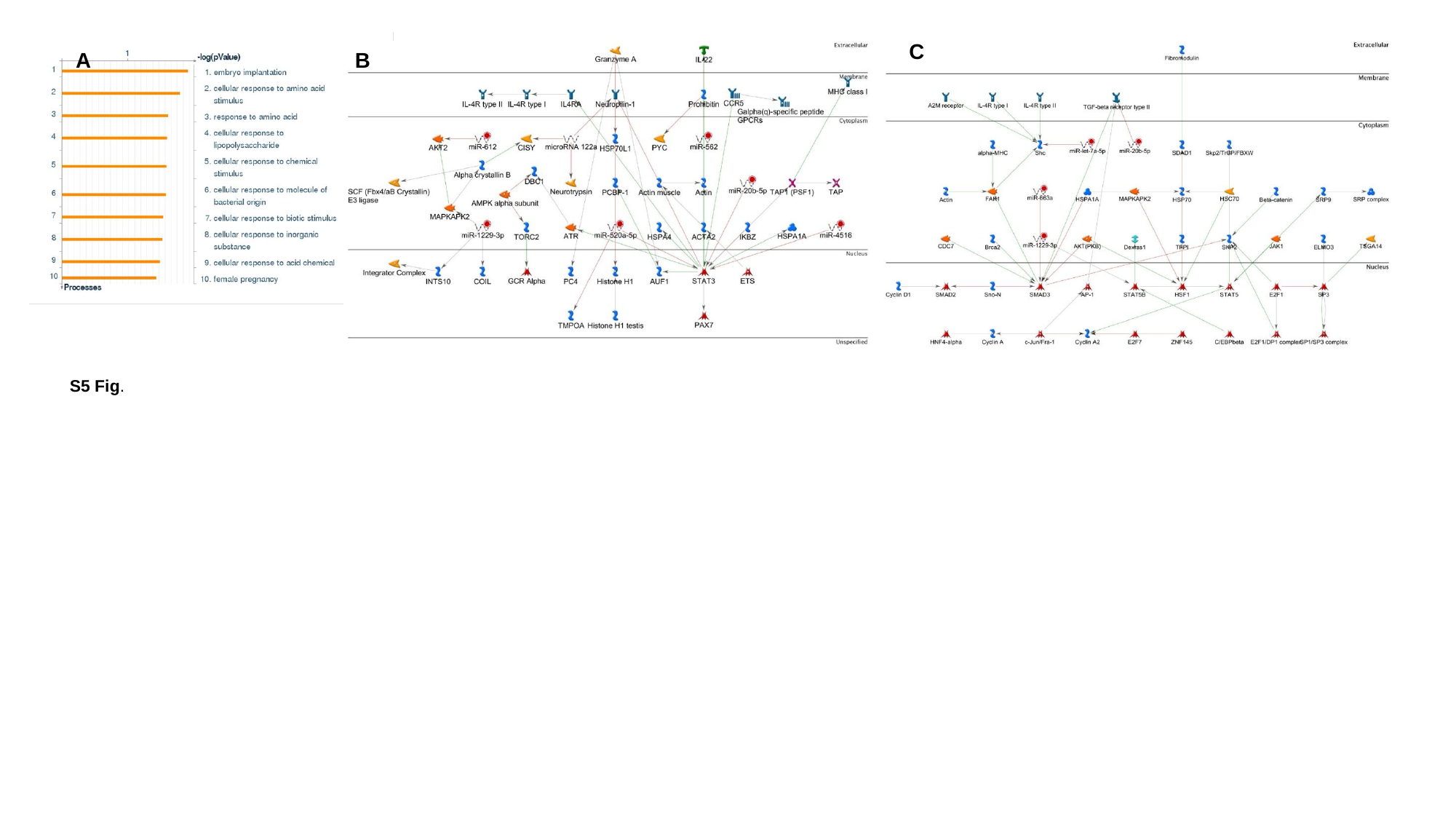

C
A
B
S5 Fig.

Supplement: S5 Fig — A) Gene Ontology (GO) processes. MetaCore Analyze Networks algorithm identified B) miR 1229 3p, miR 520a 5p, miR 612, miR 4516, miR 562: positive regulation of metabolic process (60.5%), negative regulation of apoptotic process (37.2%), negative regulation of programmed cell death (37.2%), negative regulation of cell death (37.2%), viral process (34.9%); C) miR 20b 5p, miR 663a, miR let 7a 5p, miR 1229 3p, SMAD3: regulation of cell proliferation (65.2%), cellular response to growth factor stimulus (43.5%), response to growth factor (43.5%), positive regulation of macromolecule metabolic process (71.7%), response to lipid (52.2%) (PPTX) [file pone.0184471.s005.pptx]

## Slide 1
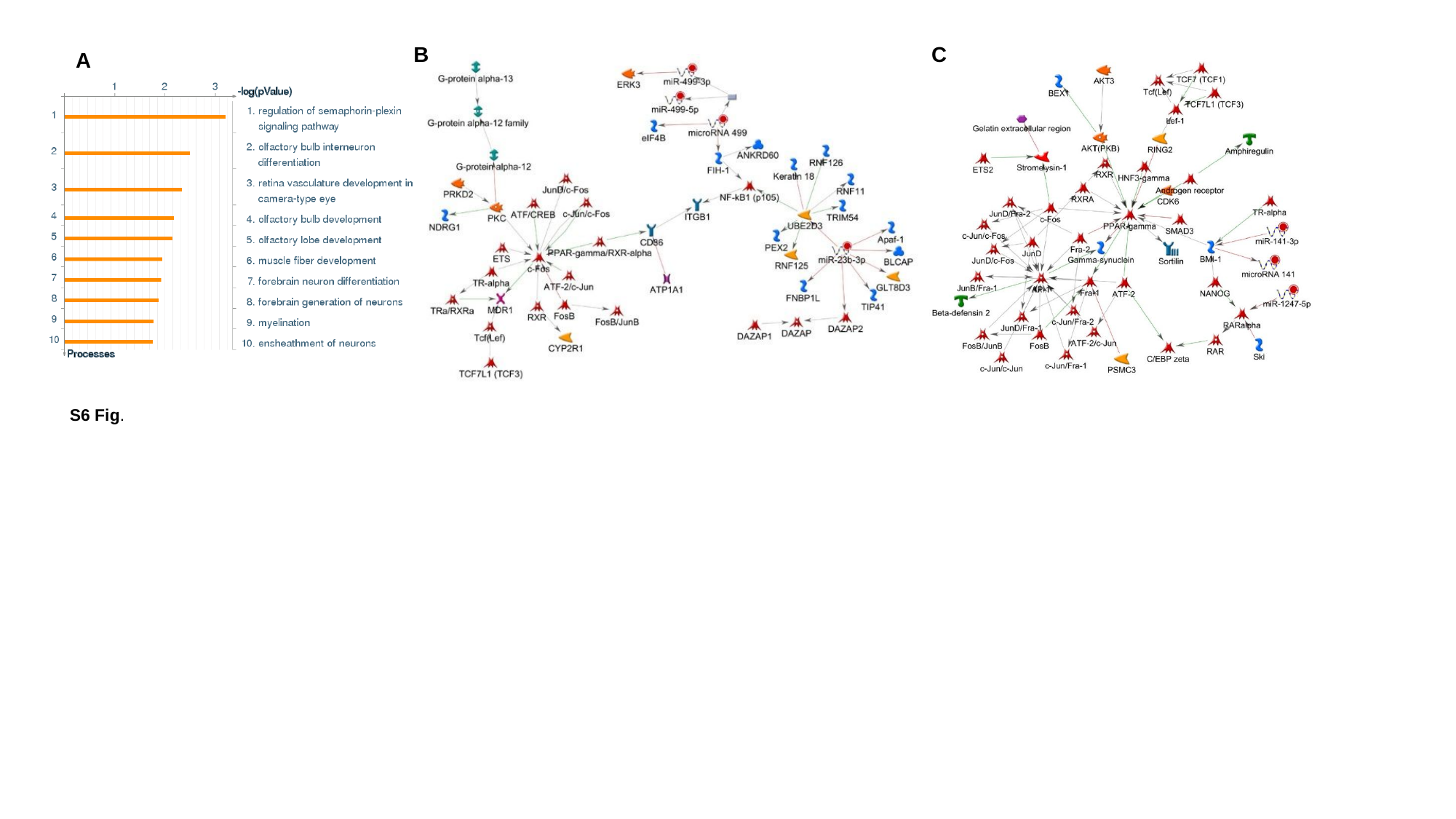

C
B
A
S6 Fig.

Supplement: S6 Fig — A) Gene Ontology (GO) processes. MetaCore Analyze Networks algorithm identified B) miR-23b-3p, miR-499, miR-499-3p, miR-499-5p, c-Fos: response to drug (37.8%), response to abiotic stimulus (48.9%), response to mechanical stimulus (28.9%), cellular response to hormone stimulus (37.8%), response to inorganic substance (37.8%). C) miR-141, miR-141-3p, miR-1247-5p, PPAR-gamma, BMI-1: positive regulation of transcription from RNA polymerase II promoter (76.6%), regulation of transcription from RNA polymerase II promoter (85.1%), positive regulation of nucleic acid-templated transcription (76.6%), positive regulation of transcription, DNA-templated (76.6%), negative regulation of RNA metabolic process (74.5%). (PPTX) [file pone.0184471.s006.pptx]

## Slide 1
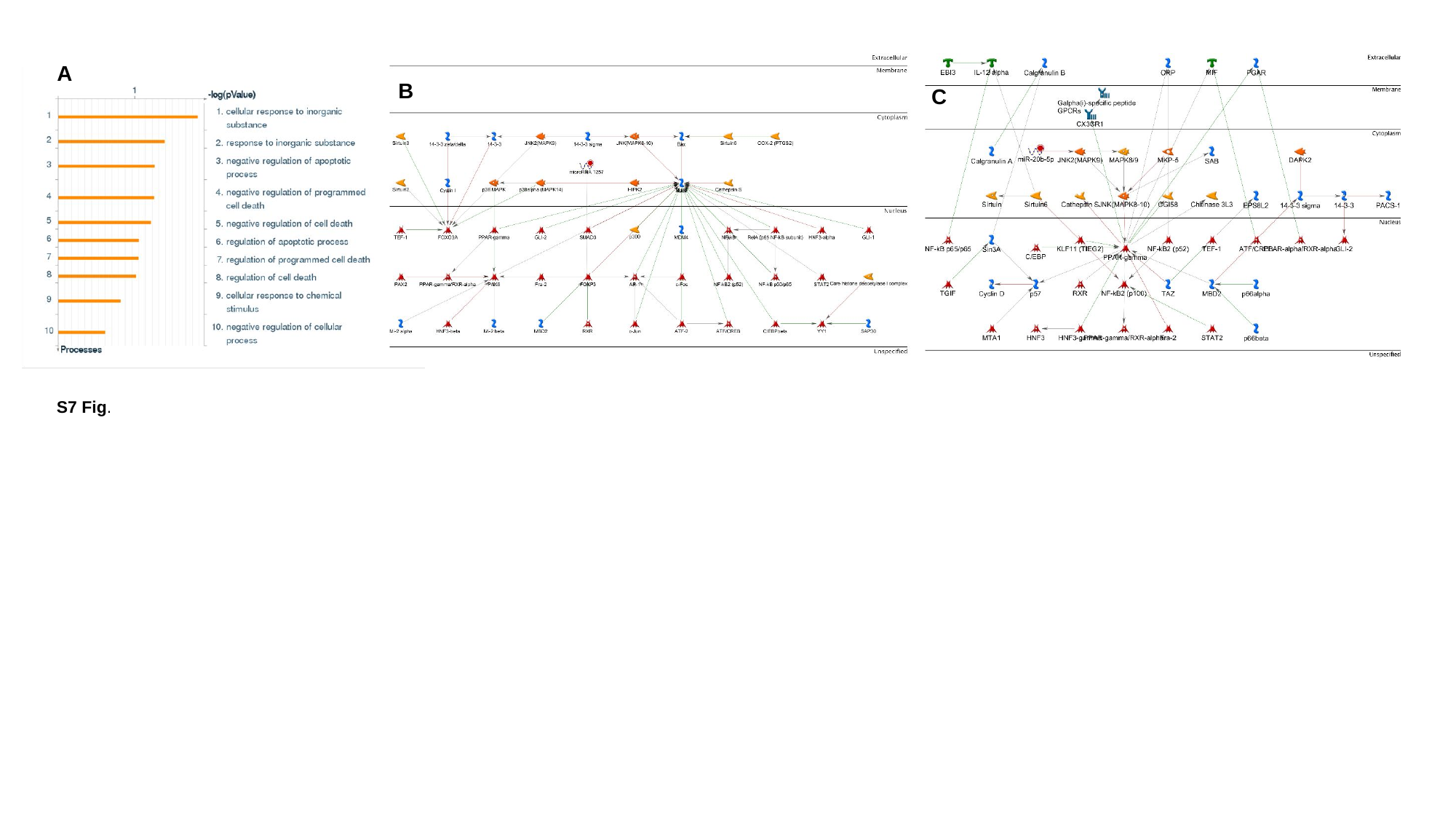

A
B
C
S7 Fig.

Supplement: S7 Fig — A) Gene Ontology (GO) processes. MetaCore Analyze Networks algorithm identified B) miR-1257, Bcl-2, PAX6, FOXO3A, and FOXP3; and C) miR-20b-5p, PPARγ, MDA2, p57, Sin3. (PPTX) [file pone.0184471.s007.pptx]
